# Beaver Fever: Whole-Genome Characterization of Waterborne Outbreak and Sporadic Isolates To Study the Zoonotic Transmission of Giardiasis

**DOI:** 10.1128/mSphere.00090-18

**Published:** 2018-04-25

**Authors:** Clement K.-M. Tsui, Ruth Miller, Miguel Uyaguari-Diaz, Patrick Tang, Cedric Chauve, William Hsiao, Judith Isaac-Renton, Natalie Prystajecky

**Affiliations:** a Department of Pathology and Laboratory Medicine, University of British Columbia, Vancouver, BC, Canada; b School of Population and Public Health, University of British Columbia, Vancouver, BC, Canada; c British Columbia Public Health Laboratories, Provincial Health Services Authority, Vancouver, BC, Canada; d Department of Mathematics, Simon Fraser University, Burnaby, Vancouver, BC, Canada; Stanford University

**Keywords:** WGS, amplification host, beaver, genomic epidemiology, one health, parasites, disease outbreaks, zoonotic

## Abstract

*Giardia* causes the diarrheal disease known as giardiasis; transmission through contaminated surface water is common. The protozoan parasite’s genetic diversity has major implications for human health and epidemiology. To determine the extent of transmission from wildlife through surface water, we performed whole-genome sequencing (WGS) to characterize 89 Giardia duodenalis isolates from both outbreak and sporadic infections: 29 isolates from raw surface water, 38 from humans, and 22 from veterinary sources. Using single nucleotide variants (SNVs), combined with epidemiological data, relationships contributing to zoonotic transmission were described. Two assemblages, A and B, were identified in surface water, human, and veterinary isolates. Mixes of zoonotic assemblages A and B were seen in all the community waterborne outbreaks in British Columbia (BC), Canada, studied. Assemblage A was further subdivided into assemblages A1 and A2 based on the genetic variation observed. The A1 assemblage was highly clonal; isolates of surface water, human, and veterinary origins from Canada, United States, and New Zealand clustered together with minor variation, consistent with this being a panglobal zoonotic lineage. In contrast, assemblage B isolates were variable and consisted of several clonal lineages relating to waterborne outbreaks and geographic locations. Most human infection isolates in waterborne outbreaks clustered with isolates from surface water and beavers implicated to be outbreak sources by public health. In-depth outbreak analysis demonstrated that beavers can act as amplification hosts for human infections and can act as sources of surface water contamination. It is also known that other wild and domesticated animals, as well as humans, can be sources of waterborne giardiasis. This study demonstrates the utility of WGS in furthering our understanding of *Giardia* transmission dynamics at the water-human-animal interface.

**IMPORTANCE** Giardia duodenalis causes large numbers of gastrointestinal illness in humans. Its transmission through the contaminated surface water/wildlife intersect is significant, and the water-dwelling rodents beavers have been implicated as one important reservoir. To trace human infections to their source, we used genome techniques to characterize genetic relationships among 89 *Giardia* isolates from surface water, humans, and animals. Our study showed the presence of two previously described genetic assemblages, A and B, with mixed infections detected from isolates collected during outbreaks. Study findings also showed that while assemblage A could be divided into A1 and A2, A1 showed little genetic variation among animal and human hosts in isolates collected from across the globe. Assemblage B, the most common type found in the study surface water samples, was shown to be highly variable. Our study demonstrates that the beaver is a possible source of human infections from contaminated surface water, while acknowledging that theirs is only one role in the complex cycle of zoonotic spread. Mixes of parasite groups have been detected in waterborne outbreaks. More information on *Giardia* diversity and its evolution using genomics will further the understanding of the epidemiology of spread of this disease-causing protozoan.

## Abstract (precis)

Giardia duodenalis causes large numbers of gastrointestinal illness in humans. Its transmission through the contaminated surface water/wildlife intersect is significant, and the water-dwelling rodents beavers have been implicated as one important reservoir. To trace human infections to their source, we used genome techniques to characterize genetic relationships among 89 *Giardia* isolates from surface water, humans, and animals. Our study showed the presence of two previously described genetic assemblages, A and B, with mixed infections detected from isolates collected during outbreaks. Study findings also showed that while assemblage A could be divided into A1 and A2, A1 showed little genetic variation among animal and human hosts in isolates collected from across the globe. Assemblage B, the most common type found in the study surface water samples, was shown to be highly variable. Our study demonstrates that the beaver is a possible source of human infections from contaminated surface water, while acknowledging that theirs is only one role in the complex cycle of zoonotic spread. Mixes of parasite groups have been detected in waterborne outbreaks. More information on *Giardia* diversity and its evolution using genomics will further the understanding of the epidemiology of spread of this disease-causing protozoan.

## INTRODUCTION

*Giardia* spp. are ubiquitous, flagellated, protozoan parasites that can cause gastrointestinal infections in humans and animals (1). Six *Giardia* species have been recognized. They demonstrate a spectrum of host specificity with Giardia duodenalis (syn. G. intestinalis and G. lamblia) infecting humans and other mammals, G. agilis infecting amphibians, G. ardeae and G. psittaci infecting birds, and G. muris and G. microti infecting rodents (2–4). G. duodenalis has been considered a species complex with at least eight distinct groups or assemblages (A to H) based on nucleotide or protein polymorphisms (2, 4). Assemblage A Giardia duodenalis and assemblage B, a genetically distinct zoonotic species that some refer to as G. enterica (5), are the most common species found in mammals (2, 6).

Giardia duodenalis causes large numbers of gastrointestinal infections (giardiasis, sometimes known as beaver fever) worldwide; health impacts include diarrhea, resulting in malabsorption and impaired growth in children (1). Approximately 280 million people per year are estimated to be infected globally with 1.2 million new cases reported annually in the United States (7). On average, 627 new infections are reported yearly in the western Canadian province of British Columbia (BC) (8). Given its prevalence in both the developed and developing worlds, the World Health Organization (WHO) has now included *Giardia* as one of the major food-borne pathogens (http://www.who.int/mediacentre/factsheets/fs399/en/); giardiasis has also been included in the WHO’s Neglected Diseases Initiative with the goal of providing further insight into the protozoan biology, infection epidemiology, and host-parasite interactions (9). Despite its significance, transmission is still poorly understood.

The infective form of *Giardia* is the cyst, excreted in feces from animal and human hosts and capable of persisting in the environment for months. Transmission to new hosts is by the direct person-to-person (fecal-oral) route or by the indirect route, after ingestion of contaminated food or water (1, 10–12). Spread through surface water is important in many parts of the world (1, 11, 13). Surface water may be an important factor in animal-to-human transmission and the reverse, human-to-animal spread. Since infective cysts may persist in surface water at 0 to 40°C for more than 2 months (1) and since cysts are relatively resistant to drinking water chemical treatment, this route of spread is more common where surface drinking water is not fully treated. Many reports have suggested that a variety of wild and domesticated animals can act as sources of surface water contamination (1, 2, 14). Molecular data have confirmed that *Giardia* assemblages A and B are zoonotic (14) and ubiquitous.

Among wild animals, beavers (Castor canadensis), a water-dwelling mammal, have been implicated in the waterborne transmission of giardiasis. Since beavers can excrete cysts in feces directly into surface sources of animal and human drinking water, including lakes, rivers, and streams, zoonotic transmission of *Giardia* from beavers to humans has been suggested (15). Studies have shown that the prevalence of *Giardia*-infected beavers can range between 13 and 30% (16–19). In one report, of the 299 beavers studied during a 15-month period, 14.7% (19.5% from unique sites), were infected (18). *Giardia* has also been detected in lake water samples near beaver lodges (20). There is, however, limited data at the molecular level using genomic information mapping out this parasite’s zoonotic transmission network (21). There is even less information about the species, assemblages, and genotypes found in water-dwelling beaver hosts or surface water.

Evidence to support the idea that beavers may play an important part in the zoonotic cycle of waterborne infections includes observations that nucleotide sequences of *tpi* from G. duodenalis isolates from sporadic human infections were identical to those from beaver isolates (22). In one large waterborne outbreak, Isaac-Renton et al. (10) used pulsed-field gel electrophoresis to aid the epidemiological investigation in which public health officers had determined that a beaver was the source of the outbreak. More recently, using molecular tools (PCR) and core gene sets obtained from whole-genome sequencing (WGS), *Giardia* isolates from this and other investigations across BC were studied (23).

In the current work, we identified assemblages A and B and found mixes of assemblages in waterborne outbreaks (23). In one outbreak, WGS showed A1, A2, and B assemblages, all present in an abrupt onset and well-studied community event. One isolate retrieved from the beaver considered to be the source of this waterborne outbreak was found to be infected with assemblage A1, while two human isolates (of the eight isolates available from outbreak cases) were also A1 (23). One human source outbreak isolate, however, was found to be A2. The contaminated drinking water was found on three occasions to have assemblage B isolates, as did the other five (of eight) human infections (23). In order to better understand the source of outbreak and the pattern of transmission, analysis beyond the core gene phylogeny was then conducted.

Using a library of 89 isolates and their genetic profiles obtained by WGS, it was proposed that a WGS approach could generate even more highly resolved phylogenies and provide further insight into zoonotic transmission, specifically the role of the beaver. Since many surface water isolates were also available for study, this provided a unique opportunity to characterize the genetic types of parasites found in the environment and compare them at the WGS level with human and animal source isolates from the same or different region. Our objectives were (i) to identify G. duodenalis assemblages associated with various animal hosts, waterborne outbreaks, and/or geographical localities and (ii) to investigate the transmission dynamics and genetic variability of G. duodenalis isolates within and among outbreaks. This study also demonstrated the value of WGS in examining giardiasis transmission at the water-human-animal interface considering the one health approach.

## RESULTS

**FIG 1:**
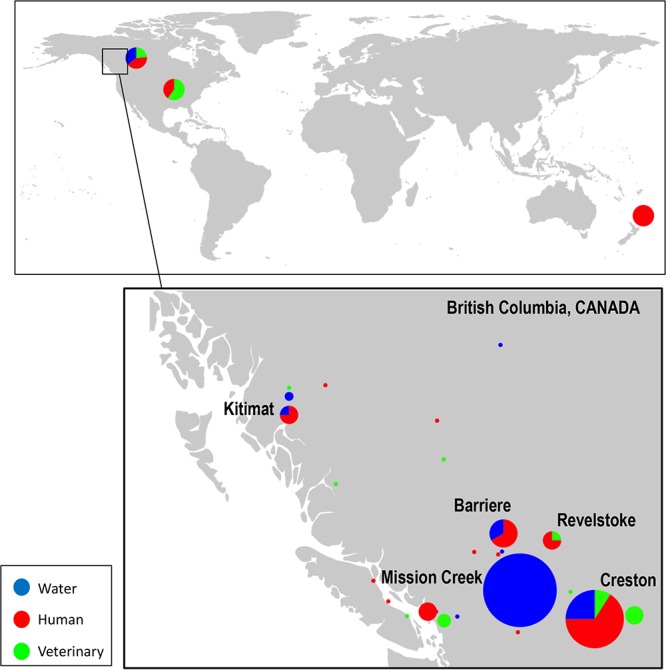
A map showing the collection of G. duodenalis isolates from this study, including waterborne giardiasis outbreaks and sporadic isolates from local and national laboratories (isolates listed in Table S1 in the supplemental material). (Map is courtesy of Jaroslav Klápště and used with permission.)

We sequenced a total of 89 *Giardia* isolate genomes, of which 29 were from raw surface water, 38 were from humans, and 22 from veterinary sources—17 from beavers, 2 from dogs, 2 from sheep, and 1 from a cat. These isolate genomes included 27 associated with waterborne outbreaks and 62 associated with sporadic human infections or retrieved from raw surface water but not associated with documented outbreaks (Fig. 1 and see Table S1 in the supplemental material). Sequencing using the Illumina MiSeq system generated 1.2 to 12 million high-quality reads for each sample. Genomic analysis by mapping to reference genomes revealed the presence of two distinct assemblages, A and B. Assemblage A could then be further divided into assemblages A1 and A2 (Table S1). The genome size of assemblage A was ca. 10 Mb, while those of assemblage B were ca. 11 Mb. Mean coverage was ca. 74× (range, 16× to 181× coverage) in each sample (Table S1). The average GC contents were 48.9% in assemblage A and 47.9% in assemblage B. On average, each assembled genome was distributed in ca. 550 contigs (>1 kb in size) with *N*_50_ values of 58,961 and 53,069 on average in assemblages A and B, respectively (Table S1).

10.1128/mSphere.00090-18.3 TABLE S1  All isolates included in the investigation with epidemiological data, assemblage, and various statistical data from the raw next-generation sequencing (NGS) reads and assembled genomes. Highlighted samples have mixed assemblages. Download TABLE S1, XLS file, 0.1 MB. © Crown copyright 2018. 2018 Crown This content is distributed under the terms of the Creative Commons Attribution 4.0 International license.

Of the 89 genomes studied, 35 were assemblage A1, 7 were A2, and 41 were assemblage B (Table 1). It was also observed that six samples contain mixed assemblages, defined as the presence of two detected assemblages—a major assemblage genotype (70 to 95% of total reads) present along with a minor assemblage genotype (3.5 to 28.7% of total reads) that accounts for more than 3% of total raw reads (Table S1). Five of these were assemblage A/B mixes, while one was an A1/A2 mix. Two samples (VANC/87/UBC/25 and VANC/90/UBC/45 [VANC stands for Vancouver, and UBC stands for University of British Columbia) also contained rather high levels of bacterial contaminations (Stenotrophomonas maltophilia and *Clostridium* spp.; 33 to 54% total reads) (Table S1). Contaminated reads were removed before further single nucleotide variant (SNV) analysis.

**TABLE 1:** Prevalence of *Giardia* assemblages in isolates grouped by source of origin

Source	Outbreak-associated isolates					Sporadic isolates					All isolates				
	*N*^a^	No. (%) of isolates in assemblage:				*N*	No. (%) of isolates in assemblage:				*N*	No. (%) of isolates in assemblage:			
		A1	A2	B	Mix		A1	A2	B	Mix		A1	A2	B	Mix
Humans	17	4 (23.5)	1 (5.9)	11 (64.7)	1 (5.9)	21	14 (66.7)	5 (23.8)	2 (9.5)		38	18 (47.4)	6 (15.8)	13 (34.2)	1 (2.6)
Water	8	3 (37.5)		5 (62.5)		21	3 (14.3)		16 (76.2)	2 (9.5)	29	6 (20.6)		21 (72.4)	2 (7)
Veterinary	2	1 (50)		1 (50)		20	10 (50)	1 (5)	6 (30)	3 (15)	22	11 (50)	1 (4.5)	7 (31.8)	3 (13.7)

Total	27	8 (29.6)	1 (3.7)	17 (63)	1 (3.7)	62	27 (43.5)	6 (9.7)	24 (38.7)	5 (8.1)	89	35 (39.3)	7 (8)	41 (46)	6 (6.7)

a *N*, number of isolates.

Of the 29 raw surface water isolates, 6 were assemblage A1, 21 were assemblage B, and 2 were mixed isolates (Table 1). Among the 38 human source isolates, 18 were assemblage A1, 6 were A2, 13 were assemblage B, and 1 was a mix. Of the 22 veterinary source isolates, 11 were assemblage A1, 1 was A2, 7 were assemblage B, and 3 were mixed. For the 17 beaver isolates, 7 belonged to assemblage A1, 1 was A2, 6 were assemblage B, and 3 were mixed (Table S1). Two dog isolates were A1 and B assemblages, respectively, while two sheep isolates and one cat isolate were assemblage A1 (Table S1). Both assemblage A and B isolates were retrieved from outbreak samples with the predominant type being assemblage B isolates (64.7% of human isolates, 62.5% in outbreak-associated surface water) (Table  2). In samples not associated with outbreaks, assemblage A1 (66.7%) and A2 (23.8%) were more prevalent in human source isolates. The majority (76.2%) of non-outbreak-associated surface water isolates were assemblage B in this study (Table 1).

**TABLE 2:** *Giardia* isolates from waterborne outbreaks in Canada summarized by assemblage

Town with outbreak	Isolate source (*n*)^a^	Collection date	Assemblage(s) (*n*)
Creston, BC	Drinking water (3)	1990 February-March	B (3)
	Beaver (1)	1990 March	A1 (1)
	Human (8)	1990 February	B (5), A1 (2), A2 (1)

Kitimat, BC	Drinking water (1)	1990 March	B (1)
	Human (3)	1989 December, 1990 February	A1 (1), B (2)

Barriere, BC	Drinking water (2)	1990 July, September	A1 (1), B (1)
	Human (3)	1990 December, 1991 January	A1 (1), B (2)

Revelstoke, BC	Human (3)	1995 September	Mix A1/B (1), B (2)
	Beaver (1)	1995 August	B (1)
Botwood, NL	Water (2)	1993 June	A1 (2)

a *n*, number of isolates.

**FIG 2:**
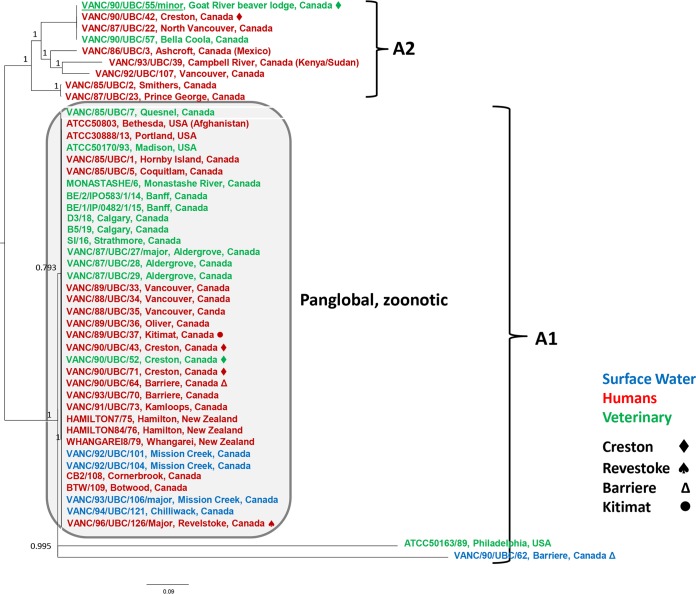
Maximum likelihood phylogeny of whole-genome SNV for G. duodenalis assemblage A isolates with 500 bootstrap replicates. Isolates are colored according to their source (water [blue], human [red], and veterinary [green]).

**FIG 3:**
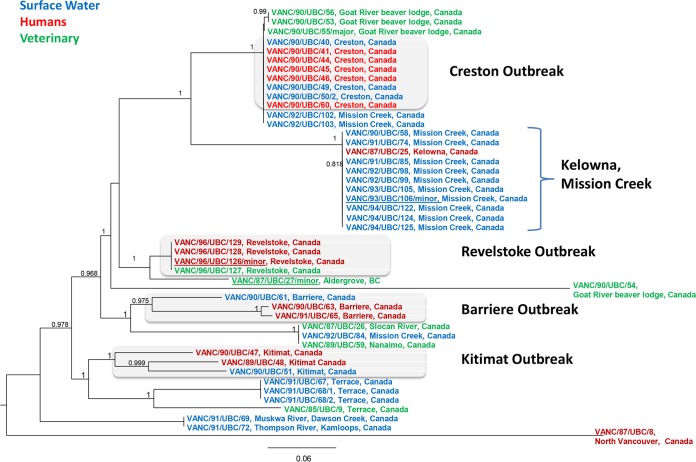
Maximum likelihood phylogeny of whole-genome SNPs for Giardia duodenalis assemblage B isolates with 500 bootstrap replicates. Isolates are colored according to their source (water [blue], human [red], and veterinary [green]).

Five samples (VANC/87/UBC/27, VANC/90/UBC/55, VANC/93/UBC/106, VANC/94/UBC/121, and VANC/96/UBC/126) were found to have mixed A/B assemblages (Table S1). Of these five samples, in addition to the contigs from the major assemblages, we also included the contigs assembled from reads associated with the minor assemblages from four samples (VANC/87/UBC/27, VANC/90/UBC/55, VANC/94/UBC/121, and VANC/96/UBC/126) in the final SNV analysis. Thus, in total, 93 isolates were included in the genomic analysis represented in the SNV trees (89 samples with majority assemblage, plus 4 minority assemblage genotypes from the mixed samples) (Fig. 2 and 3). We were unable to include the minor assemblage from sample A1/A2 (VANC/85/UBC/7) mix due to the computational challenge of filtering the reads of A2 from A1. Also one A1/B mix sample (VANC/94/UBC/121) had only 3% reads mapped to the minor assemblage; thus, the minor assemblage was not included in the final SNV analysis. After filtering for low-quality SNVs, phylogenetic trees inferred from the remaining high-quality SNVs were plotted separately for assemblages A and B due to their genomic divergence (Fig. 2 and 3).

Forty-seven isolates were included in the SNV tree (17,920 SNVs) for assemblage A, including the minor assemblage A2 genotype from isolate VANC/90/UBC/55 (Fig. 2). Thirty-eight assemblage A1 isolates, including water, human, and veterinary sources from Canada, United States, and New Zealand, clustered together with strong statistical support (Fig. 2); most isolates in assemblage A1 were very clonal (99.89 to 100% SNV similarity), with the exception of two divergent isolates—ATCC 50163 from a cat and an isolate from a surface water sample (VANC/90/UBC/62) from Canada (ca. 56% and 52% similarity to other A1 isolates). No significant SNV variation among 36 assemblage A1 isolates was observed when geographical source was considered; isolates from Canada and United States, as well as from New Zealand, clustered together. They are referred to as the panglobal, zoonotic isolates (Fig. 2). Veterinary A1 isolates from dogs, beavers, and sheep clustered together with no discernible high-quality SNVs, except for one beaver isolate from northern British Columbia (raw data indicated mix A1/A2 assemblage). The genomes of isolates from various outbreaks and sporadic collections were also >99% identical to each other during contig alignment (Table S2).

10.1128/mSphere.00090-18.4 TABLE S2  Genomic similarity among all samples (assemblages A and B) using MUMmer. Download TABLE S2, XLS file, 0.1 MB. © Crown copyright 2018. 2018 Crown This content is distributed under the terms of the Creative Commons Attribution 4.0 International license.

In contrast to the highly clonal assemblage A1 isolates, assemblage A2 isolates were relatively variable compared to each other (85.74 to 100% SNV similarity) though they shared 94% similarity at the level of contig alignment to A1 (Fig. 2 and Table S2). Three major clusters were found among the assemblage A2 isolates studied. In the overall analysis, two isolates from patients living in northern BC with no travel history outside this province and retrieved 2 years apart clustered together. Two isolates from patients (VANC/92/UBC/107 and VANC/93/UBC/39) with travel histories to Sudan/Kenya and Mexico, respectively, were more divergent from other A2 isolates but clustered with an isolate from a 4-year-old BC human without travel history (VANC/86/UBC/3) (Fig. 2 and Table S1).

All isolates of assemblage B in this study were retrieved from BC sources. They were found to be highly divergent (ca. 47% to 100% SNV similarity) based on more than 14,399 SNVs across the genome. These isolates could be divided into various well-supported (>90% bootstrap support) groups/lineages (Fig. 3). All outbreak isolates were closely related to other isolates from the same outbreak in the whole-genome SNV-based phylogenetic tree. This finding is consistent with epidemiological data from public health investigations (Fig. 3 and Table S1). The inferred phylogeny of assemblage B isolates showed that most outbreaks were independent and involved genetically distinct isolates (Fig. 3). A positive correlation (*r*^2^ = 0.16; *P* = 0.01) was also established between the pairwise geographic distance of the locations and the genetic distance among the assemblage B isolates, suggesting that different community outbreak isolates were not linked; in each outbreak, there was local transmission (Fig. S1).

10.1128/mSphere.00090-18.1 FIG S1  Correlation between pairwise genetic distance (SNVs) and geographical distance in Giardia duodenalis assemblage B isolates. Download FIG S1, TIF file, 0.1 MB. © Crown copyright 2018. 2018 Crown This content is distributed under the terms of the Creative Commons Attribution 4.0 International license.

**FIG 4:**
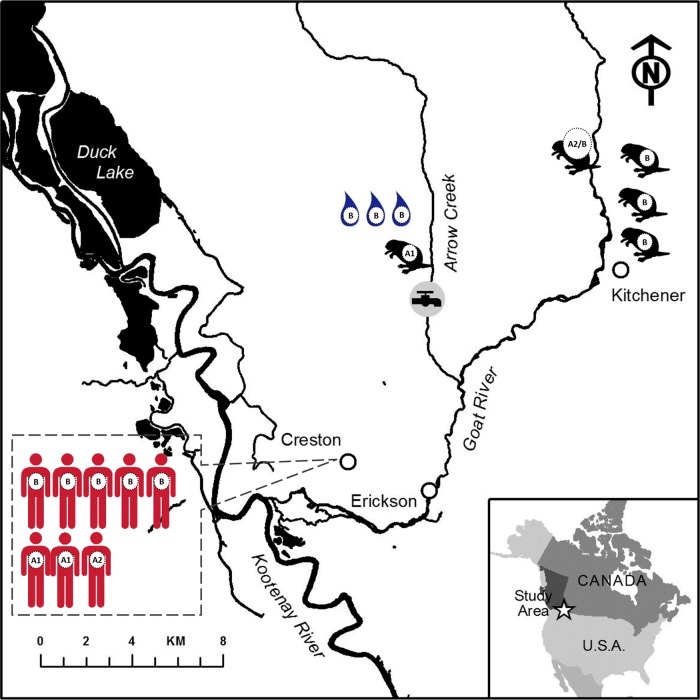
A recapitulation of the Creston waterborne outbreak in Canada based on the genomic SNV data in this study. (Map is courtesy of Sunny Mak and used with permission.)

Based on the SNV tree of assemblage B and percent contig similarity among assembled genomes based on MUMmer (Table S2), the outbreak-associated assemblage B isolates of human sources were clonal and always clustered with water or beaver source isolates, with strong statistical support for each outbreak (similarity between contigs ranged from 99% in the Creston waterborne outbreak to 96 to 99% in the Barriere outbreak) (Table S2). For instance, three assemblage B isolates from the Barriere outbreak (two isolates from humans and one isolate from water) clustered together. Another three assemblage B isolates from the Kitimat waterborne outbreak also clustered together (Fig. 3). Four assemblage B isolates (one isolate from a beaver and three isolates from humans) from the Revelstoke waterborne outbreak were 100% identical to each other at the genomic level. Eight assemblage B isolates (five isolates from humans and three from surface drinking water) from the Creston outbreak clustered into a single group. These isolates also grouped with three “sporadic” beaver isolates collected after the outbreak from a beaver group (lodge) found in a nearby but separate river, the Goat River beaver lodge, ca. 11  km east of Creston (Fig. 4). The assemblage B isolates from three of the four beavers from the Goat River beaver lodge clustered with the Creston outbreak isolates; these three beaver isolate genotypes originated from the water and human sources based on SNV inference. We also note that one of the four beaver isolates (VANC/90/UBC/54) from the Goat River group was infected with an assemblage B genotype that was divergent from the other three Goat River assemblage B beaver isolates.

Both SNV trees showed that each of the BC waterborne outbreaks included both assemblage A and B isolates (Table 2 and Fig. 2 and 3). In the Creston outbreak, 12 samples included assemblages A1, A2, and B. The other three outbreaks, Kitimat, Barriere, and Revelstoke, had both A1 and B assemblages (Table 2 and Fig. 2 and 3). Most outbreak-associated water isolates from widely separated regions of BC had assemblage B genotypes (Table 1).

In surface water samples collected from Mission Creek over a 4-year period, no assemblage A2 was observed; both assemblage A1 (2 of 16) and B (13 of 16) and a mix were detected (Table 3). In this same-site, surface water group of isolates, three different assemblage B genotypes were observed over time (Fig. 3 and Table 3). In addition, one human (with no travel history) residing in Kelowna, BC, a municipality adjacent to this river (VANC/87/UBC/25) had a strong association with the 10 water samples of the Mission Creek water source collected between 1990 and 1994; they clustered as a single group with strong statistical support (Fig. 3).

**TABLE 3:** Fifteen *Giardia* isolates from surface drinking water at Mission Creek, British Columbia, Canada, studied over time and summarized by assemblage

Source and collection date	Sample	Assemblage	Genomic cluster on SNP tree^a^
Mission Creek intake			
1990 September	VANC/90/UBC/58	B	MC
1991 May	VANC/91/UBC/85	B	MC
1991 December	VANC/91/UBC/74	B	MC
1992 April	VANC/92/UBC/84	B	With dog and beaver
1992 July	VANC/92/UBC/98	B	MC
1992 September	VANC/92/UBC/101	A1	Panglobal
1992 October	VANC/92/UBC/103	B	Creston outbreak
1992 November	VANC/92/UBC/104	A1	Panglobal
1993 September (sampled twice)	VANC/93/UBC/105	B	MC
	VANC/93/UBC/106	Mix A1/B	Panglobal

Downstream of Mission Creek intake after reservoir pond			
1992 September	VANC/92/UBC/99	B	MC
1993 September	VANC/92/UBC/102	B	Creston outbreak

Downstream of Mission Creek at second pond (reservoir) inlet			
1994 August	VANC/94/UBC/122	B	MC
1994 September	VANC/94/UBC/124	B	MC
1994 October	VANC/94/UBC/125	B	MC

a MC, Mission Creek.

Although there was high diversity among BC isolates in assemblage B, our data also indicated the similarity of some assemblage B isolates collected from widely separated locations. For instance, two isolates collected from water in Mission Creek in the interior of BC in 1992 nested within the cluster of isolates from the eastern part of the province (Creston from 1990)—a distance of ca. 230 km (Fig. 3). Another water sample from interior BC (Mission Creek) clustered with an isolate retrieved from a symptomatic dog in Nanaimo, Vancouver Island in far-western BC (ca. 320 km) and a beaver isolate collected in the southwest (Slocan, BC)—again a separation geographically of ca. 150 km from Mission Creek. We also noted that an isolate from the Muskwa Creek, a water source isolate collected close to the drinking water intake for Dawson Creek, BC, clustered with an isolate from Thompson River, which was close to the water intake for another town, Kamloops (ca. 820 km from Dawson Creek) (Fig. 1 and 3).

To assess whether recombination could play a role in the *Giardia* evolution and adaptation, SplitsTree was used to generate the phylogenetic network, which also shared identical clustering pattern among isolates with FastTree. The network indicated the presence of intra-assemblage recombination in assemblage A1, A2, as well as among B isolates (Fig. S2 and S3); however, the evidence was insignificant based on Φ-test (*P* = 0.07 and 0.98 in assemblage A and B, respectively), suggesting that recombination events did not affect the interpretation of our data.

10.1128/mSphere.00090-18.2 FIG S2  Neighbor net analysis based on genome-wide SNVs showing the genetic relationships of Giardia duodenalis assemblage A (a) and assemblage B (b). Isolates are colored according to their source (water [blue], human [red], and veterinary [green]). Download FIG S2, PPT file, 0.2 MB. © Crown copyright 2018. 2018 Crown This content is distributed under the terms of the Creative Commons Attribution 4.0 International license.

## DISCUSSION

### Zoonotic transmission.

Isolates from assemblages A1, A2, and B from human and domestic veterinary sources have previously been examined using WGS, including assemblage A1 and A2 from Sweden and Norway (5, 23–26), assemblage B from Alaska, United States, and Norway (5, 27), and one assemblage E isolate from a piglet in the Czech Republic (28). However, the current study is the first to sequence the whole genome of G. duodenalis isolates retrieved from geographically and epidemiologically linked surface drinking water, human, and animal (including wildlife) sources. It is also the first study to use WGS to analyze surface water source isolates from one site collected over time. Waterborne giardiasis outbreaks occurred in widely separated geographic locations in this large Canadian province; some were clearly epidemiologically linked to untreated surface water contaminated by beavers. This study also examined water, human, and beaver isolates from these outbreaks, with a view to the one health approach.

In a past study, 12 G. duodenalis isolates from beaver fecal samples collected from six different riverbank sites in southern Alberta, Canada, were found to belong to assemblage B based on sequences of 18S rDNA (29). Beavers from Massachusetts and Maryland in the United States were also confirmed to carry assemblage B genotypes based on multilocus sequences (22, 30). The current study provides clear genomic evidence supporting the role of beavers epidemiologically identified as the cause of two small community waterborne outbreaks (10). One beaver (VANC/90/UBC/52) removed from near the Arrow Creek (cyst-contaminated drinking water) drinking water intake during the Creston outbreak exhibited the same assemblage A1 genotype as two human isolates retrieved during the outbreak (Fig. 2 and 4). Similarly, another beaver isolate (VANC/90/UBC/55) trapped in a Goat River beaver lodge, which was not associated directly with the outbreak, had A2/B infections with a majority of the same assemblage B (also with minority assemblage A2 genotype shared by another Creston outbreak clinical isolate [VANC/90/UBC/42]) (Fig. 3 and 4). In addition, isolates from two other beavers from the Goat River beaver lodge (as noted, not directly associated with the Creston outbreak but located in a river about 10 km from Creston, near Kitchener, BC) clustered with the three water system isolates retrieved from the contaminated water source (Arrow Creek) causing the Creston outbreak and with five human isolates retrieved during the outbreak investigation (Fig. 4). These isolates all shared >98% identity in SNVs and genomic content. We note that Arrow Creek and Goat River are connected some distance downstream of the drinking water intake. We also note that, since juvenile beavers are able to migrate for long distances in the watershed when pressured, it is possible that the isolate from the lone beaver living under the snow and ice at the drinking water intake in Arrow Creek originated from the nearby Goat River beaver colony (10). Similarly, in the Revelstoke outbreak, the beaver infected by an assemblage B genotype and residing in the culvert at the community drinking water intake, and considered by public health to be the cause of the outbreak, was identical at the whole-genome SNV level of that of the three outbreak-associated human isolates (Fig. 3). This is consistent with the results based on PCR-restriction fragment length polymorphism (RFLP) of *tpi* in these Revelstoke isolates (31). In both Creston and Revelstoke waterborne outbreaks, the drinking water source was upstream of the town and in remote areas without a human residence or the presence of domestic animals (18, 31). The epidemiological conclusions from the two giardiasis outbreaks are consistent with current SNV findings suggesting that beaver-to-human spread is important and that beavers were the source of human infections in these events. We do not, however, rule out the possibility of human influence on infections of beavers (human-animal-human dynamic) overall.

**FIG 5:**
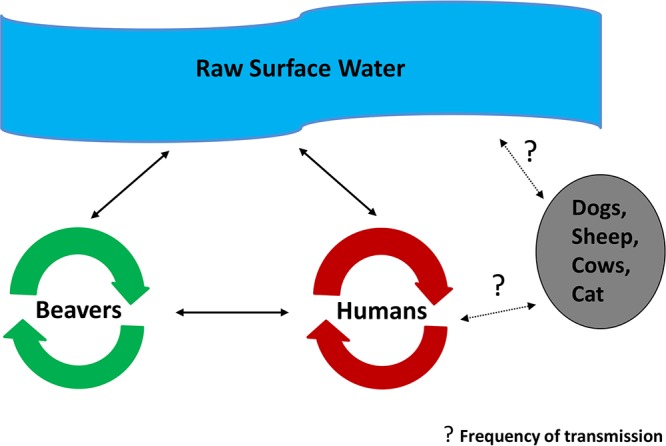
Proposed scheme for the zoonotic cycle of transmission in giardiasis.

Our findings are consistent with the proposed role of beavers as an amplification or reservoir host (14, 32) for waterborne spread of zoonotic G. duodenalis species (Fig. 5). Increased numbers of cysts from amplification hosts that reside in surface water supplies with ongoing contamination of water downstream has been shown to relate to beaver breeding activities in the late spring and early fall activities preparing for winter (33, 34). Thus, nonimmune beavers exposed to cysts from humans or other animal hosts contaminating the surface water in which they live, when infected with a zoonotic species, could produce ongoing contamination of the water source (Fig. 5). As G. duodenalis trophozoites proliferate in the intestinal tracts of new beaver hosts, more cysts are excreted into their water, perpetuating contamination particularly during spring and early fall activities, hence their role in giardiasis transmission, acting as an infection amplification host for zoonotic species (14, 32, 34, 35). This role is seen more clearly when beaver infections are spread to humans (zoonotic species) in outbreak events, particularly when these outbreaks are associated with watersheds with limited domestic animal or human activities, such as two of the BC events studied.

We also note that three other beavers (one with mixed assemblage isolates) from a beaver lodge near Aldergrove, a southeastern BC town, were infected with identical A1 genotypes. Since assemblage A1 is lacking in genetic variability, less can be inferred about their genetic relatedness. In a similar fashion to the Goat River beaver lodge group, since the beavers live together in the same contaminated water source, highly genetically related isolates from beaver lodge members is not unexpected. It is possible that, similar to human-to-human spread (1, 2) reported in settings such as child day care centers, animal-to-animal spread within the beaver lodge also occurs (19) (Fig. 5). Another possibility is that, since one beaver in the Goat River beaver group showed a divergent isolate compared to the assemblage B isolates of others, the Aldergrove beaver lodge had different types of infections, but this was not detected either because of the clonality of assemblage A1 (Fig. 4 and 5) or because of the bias inherent to culture-based isolate retrieval (36).

Although no beaver hosts were directly identified as the source of the outbreaks in Barriere and Kitimat, both watersheds providing community drinking water were known to have beavers, along with other wildlife, living in them. We note that all BC outbreak communities studied used surface water sources from watersheds with very little human influence. It is well-known, however, that many types of wildlife such as deer, wolves, voles, moose, elk, coyote, beaver, and as noted above, many kinds of domesticated animals, including dogs, cats, sheep, cattle, as well as humans may cause spread (14, 18, 37–40). Although not acting as amplification hosts, domestic animals in close contact with humans may also play an important role in human infection and environmental contamination. One study showed that G. duodenalis cysts from fecal samples from humans and dogs in a remote community in India typed at multiple genetic loci, and similar assemblages (A and B) were observed in humans and dogs in the same household (41). One of our study dog samples (Vancouver Island, Nanaimo, BC) was found to be assemblage B and clustered with a water isolate from interior BC (Mission Creek) as well as a beaver isolate (not associated with an outbreak) from northern BC. The second dog sample from Calgary, Alberta, Canada, was found to have an A1 genotype and clustered with other human, sheep, and water isolates collected from sources across Canada, United States, and New Zealand. Thus, animal-to-human transmission is clearly possible. A systematic review of giardiasis in Brazil also suggested that dogs and cats could be disseminators of *Giardia*, but at the same time, the results indicated that humans were a major source of transmission (40).

The SNV-based clustering of an assemblage B human isolate (collected in 1987 from an ill patient residing in Kelowna), with 10 assemblage B water isolates (collected between 1990 and 1994 from Mission Creek, 15 km east of Kelowna [Fig. 1]) could again be considered in two ways with regard to the original source of watershed contamination. First, humans infected with zoonotic species contaminate the environment, and then wildlife or domestic animals amplify and continue to circulate the parasite in that watershed (14). In this example, the resident of the nearby town (Kelowna) could have contaminated the Mission Creek watershed (a rural area with minimal human habitation) with ongoing watershed contamination observed (over a 4-year study period). Thus, a zoonotic *Giardia* assemblage B was maintained, circulating among animals living in this watershed and resulting in ongoing surface water contamination. Alternatively, it was also possible that this assemblage B genotype originated from and was maintained by animal hosts in this watershed over time with the patient from Kelowna, BC (near Mission Creek) becoming infected by exposure to Mission Creek surface water. We also note that the several different assemblages and SNV genotypes within assemblage B, all from one surface water body studied over years (Table 3), are consistent with multiple sources of parasites contaminating this river (42, 43).

Cattle are another source of zoonotic spread of G. duodenalis through surface water (44). It was observed that higher concentrations of *Giardia* cysts were detected in Mission Creek during peak calving time (45). The prevalence of giardiasis in BC cattle has been reported as ranging from 16.7% to 70% in six locations from four watersheds (46, 47). It is also known that although G. duodenalis assemblage A and assemblage B have both been detected in cattle, nonzoonotic assemblage E is the predominant assemblage in most studies, including those of western Canada (1, 2, 48). No assemblage E isolates, however, were detected in our study (42).

### Mixed infections and tools for further study.

Our data demonstrated the occurrence of mixed assemblages during waterborne outbreaks. Direct evidence for mixed infections in beavers (two beaver infections, one from an Aldergrove pond beaver lodge and one from a Goat River beaver lodge) are reported for the first time. Considering the Creston outbreak with only a single possible source of the deep winter waterborne outbreak, this study also provides indirect evidence of a mixed infection/coinfection occurring in the single (beaver source) host. While this observation has not been made previously in any other giardiasis outbreak (42, 49), infections with mixed assemblages have been reported in both humans and animals (1, 2, 50). The overall indirect evidence for this includes the following: this was an abrupt onset, well-defined outbreak; some isolates from humans infected during the outbreak exhibited assemblages different than those of beavers but with the same assemblages as those from the contaminated drinking water, and mixes were well documented. Thus, given the short-lived and explosive outbreak with a single source of contamination, we suspect that the beaver in Arrow Creek was infected with assemblages A1, A2, and B. We suggest that mixed assemblages in host and environmental samples could be more common than currently thought. It is probable that mixes are underestimated even in the current study, since genetic evidence may have been lost during isolate retrieval and culture.

We note that genotyping by conventional PCR and sequencing of a single locus may produce preferential amplification of one assemblage over another assemblage, also resulting in an underestimate of mixes (2, 49). Whole-genome SNV phylogeny may offer an advantage, in this regard, over other genotyping methods, with a better ability to detect mixed assemblages and having better ability to generate higher-resolution isolate characterization. At the same time, it is noted that the research method used in the current study has its own biases (primarily related to trophozoite propagation as noted above). For example, the lack of assemblages C, D, and E detected in animal samples and the relatively low proportion of assemblage A2 in the study collection (51) may be based on the phenotypic ability of the isolates to adapt to *in vitro* growth.

Reference genome selection may also bias SNV analysis particularly when query genomes are of variable evolutionary distances from the reference genome used and when the reference genome is not completely sequenced (52) (for example, the reference genome in assemblage B was incomplete). Since our data set represents the largest collection of *Giardia* genomic sequences thus far, instead of using a single published reference genome for SNV analysis, we used a reference-free SNV analysis algorithm and constructed assemblages A and B specific pan-genomes as the reference. This allowed direct comparison of isolates without using a reference genome as an intermediate. Such an approach has the same sensitivity as the mapping-based approach (53, 54), while including pan-genome lineage-specific regions not present in the published reference genome.

Genomic data confirmed that assemblages A1 and B of G. duodenalis are widespread in water, human, and veterinary source samples in British Columbia, Canada. The predominance of assemblage A1 in sporadic human infections in the current study is consistent with other studies, although assemblages A2 and B were also observed to cause some human infections (1, 55). No other assemblages were detected in this large number of water, veterinary, and human isolates studied. No unique, adapted assemblage was detected in beavers; assemblage B has been noted to be prevalent in previous reports (1), while A1 and A2 have been rarely reported in beavers (15, 56).

The genetic divergence between assemblages A and B is congruent with the theory that assemblages A and B are in fact two distinct biological species (1, 5, 27). The lack of evidence for interassemblage gene flow and recombination suggests that these two assemblages can be reproductively isolated (57). The majority of assemblage A1 isolates have been reported to be highly clonal, consistent with our finding; no substructuring and genetic variability from isolates obtained from human, beaver, sheep, or dog sources was observed. This suggests that assemblage A1 is a highly adapted, panglobal, zoonotic genotype (1). The genetic variability in the samples from surface water (VANC/90/UBC/62) and a cat (ATCC 50163) may reflect specific local adaptations. Assemblage A2 has been reported from humans and domesticated animals, such as cattle, horses, dogs, and cats (1, 49). Assemblage A2 isolates are genetically and biologically more variable than A1 isolates. It was impossible, however, to assign our A2 isolates to a meaningful subcluster because of the small number of isolates available. Our findings also revealed assemblage A2 to be rather distant from A1 based on percent SNV variation. Ankarklev et al. (25) estimated the genomic divergence between strain WB and two assemblage A2 isolates to be 1% based on 100,000 SNVs; however, the current study demonstrated greater genetic divergence (ca. 15 to 20%) between assemblages A1 and A2. The divergence between assemblages A1 and A2 may be underestimated in the former study, as the two A2 samples shared only 57,680 single nucleotide polymorphisms (SNPs), while each differed from the reference genome WB by 93,900 and 100,273 SNPs, respectively (25). Differences in the sources of the samples, sequencing technology, variant calling strategies, and coverage are also possible factors contributing to disparity in heterogeneity estimates (52).

While it has been reported that assemblage B undergoes frequent recombination events, the assemblage B isolates of surface water, human, and beaver origins in the Creston and Revelstoke outbreaks, as well as the assemblage B surface water isolates in the region of Mission Creek, clustered together with high genetic similarity. This can be attributed to asexual reproduction and clonal population expansion (35, 58). Genetic heterogeneity may be driven by pressures from the host immune systems (59). On the other hand, greater genetic variation (longer branch length) was detected between isolates in the Barriere and Kitimat outbreaks based on WGS SNVs. This may indicate different selective pressures within or among hosts or a rapid microevolution between different subtypes of assemblage B (leading to the genetic differences detected).

Assemblage B, the predominant assemblage from surface water samples collected in different regions across BC, also varied genetically; thus, geographical or environmental barriers could be driving such genetic divergence. It is possible that different groups or lineages of this parasite diverged or evolved in geographic isolation due to the lack of recombination or gene flow among localized parasite population types (58). It is important to continue to investigate genomic variation in isolates from the different geographical regions.

Detecting mixed Giardia duodenalis infections is important to public health epidemiology because it may have significant clinical implication in the treatment of patients. Our study showed that multiple *Giardia* genotypes can occur within an outbreak. We also showed that different genotypes may be found in the same surface water source studied over time. Recent *in vitro* studies indicated that this parasite appears to have the capacity to modulate host immune responses and, pertinent to the unique observations from community outbreak events, that mixed infections of different assemblages or genotypes can cause enhanced intestinal cell damage (59, 60). The importance of mixes, which are probably underestimated, warrants further study. While we note that population-level “virulence” as expressed by outbreak events, is clearly related to lack of herd immunity (61), further study of intra-assemblage genetic variation and interassemblage interactions along with an exploration of the as yet unexplained species-level, polyparasite host interaction (possibly producing increased virulence), is needed (61). Better methods for optimal identification of mixed infections, in single hosts and in outbreak events, are also needed.

Human health is closely linked to the health of animals and our environment. The growing understanding of the complex ecology of G. duodenalis continues to reinforce the fundamental need for multiple layers of protection of surface water supplies used for drinking water (including watershed protection and management as well as adequate treatment). This reinforces the need for adequate treatment for small communities. The small rural BC communities impacted by the historical outbreaks described have gone on to make drinking water protection and treatment improvements. The unique niche of aquatic mammals, such as beavers in the amplification of G. duodenalis, also requires recognition for its part in the zoonotic cycle of transmission.

## MATERIALS AND METHODS

### Isolate information.

A total of 89 isolates were included in whole-genome sequencing (WGS) and single nucleotide variant analyses (see Table S1 for a complete list of isolates and their sources). All isolates used in this study are part of an archive of *Giardia* isolates housed at the British Columbia Centre for Disease Control Public Health Laboratory (BCCDC PHL). They were collected from 1985 to 1996 with appropriate human and animal ethics protocols from the University of British Columbia. Additional ethics approval was not sought for this study, as these isolates were archival. Most isolates in this study have previously been characterized using pulsed-field gel electrophoresis, PCR-based fingerprinting, and/or Sanger sequencing methods (10, 18, 42, 43).

As the investigation of the related samples from the outbreak in Creston, British Columbia (BC), Canada, were important to this study, further details of this outbreak are available (10, 23). Since isolates from the Mission Creek surface water supply were the largest number of isolates (from one single site), further information is briefly described (Table 1 and Table S1). Raw water samples were collected over 4 years, during the same overall 10-year isolate collection period as the others in this library. Water samples were collected to determine the frequency of parasite contamination and to assess the effects of settling ponds (reservoirs) on *Giardia* cyst concentration (45) (Fig. 1). Ten isolates were retrieved from raw water samples collected at the drinking water intake from Mission Creek; five additional isolates were collected from the reservoir/settling ponds and also from further down the water distribution system, after the reservoir (acting as settling ponds). Water samples were collected from all seasons over a 4-year period (Table 3). At that time, the town used settling ponds and a long contact chlorination contact time for treatment (it now fully treats water using membrane filtration and chemical disinfection). They reported no excess cases of giardiasis during the study period. Three other geographically separate surface water sites associated with outbreaks (Barriere, Kitimat, and Revelstoke) (Fig. 1) were also tested for cysts. An additional four water source isolates not associated with outbreaks were also retrieved from geographically separate water sites. No excess cases of giardiasis were reported from the four towns using these four surface drinking water supplies. Two water source isolates associated with a waterborne outbreak in another Canadian province (Newfoundland) occurring during the collection period (1991) were also included (62). Animal source isolates comprised 17 isolates from BC beavers, 2 from dogs (BC, Alberta), 2 from sheep (United States [ATCC 50170] and Canada), and 1 from a cat (United States [ATCC 50163]). These isolates were retrieved from the BCCDC PHL archive (including those provided by external research laboratories and reference collections) and are described in the studies above. We included strain WB (ATCC 50803) (assemblage A1) as the reference isolate; this isolate has been characterized as being acquired by a traveler to Afghanistan (63).

### Isolate retrieval.

*Giardia* isolates were retrieved previously by purifying cysts from surface water samples (23) and human and beaver fecal samples containing cysts (64) using both *in vitro* (65) and *in vivo* excystation methods. While previous work showed that both methods are effective (36), almost all study isolates were retrieved using the *in vivo* gerbil model, given the low numbers of cysts. All animal studies were conducted in the Faculty of Medicine animal facilities at the University of British Columbia (UBC), and animals were handled according to the guidelines and in full compliance with the UBC Animal Care and Use Program (ACUP). *Giardia* trophozoites were cultured in TYI-33 medium and cryopreserved in 10% dimethyl sulfoxide (DMSO) at −80°C (65). For the present molecular characterization study, cryopreserved trophozoites were retrieved, and DNA was obtained from the 89 study isolates as noted below.

### DNA extraction, library preparation, and whole-genome sequencing.

Genomic DNA was extracted from parasite trophozoites using a QIAamp DNA minikit (Qiagen, Mississauga, ON, Canada) with modifications. Briefly, trophozoites were centrifuged, washed with phosphate-buffered saline (PBS), and incubated at 58°C with proteinase K and buffer ATL for digestion. Extracted DNA was quantified, and its quality was assessed as described in reference 23. Paired-end (PE) DNA libraries were constructed with Nextera XT DNA kit (Illumina, San Diego, CA, USA) according to the protocols with the following modification. To remove small inserts, DNA libraries were purified twice using Ampure XP beads (Beckman-Coulter) at a 0.5× ratio. After DNA was cleaned up, traces were verified using High Sensitivity DNA chips in a 2100 BioAnalyzer (Agilent Technologies, Inc., Santa Clara, CA). Samples were then normalized, denatured, and pooled following the manufacturer’s instructions (Illumina, San Diego, CA, USA). The pooled library consisted of 6 to 10 samples (per run) (each with a unique DNA index from the Illumina Nextera Index kit) prepared as recommended by Illumina (MiSeq v2 reagent preparation guide) and loaded onto a cartridge (V2 chemistry) with 250-bp paired-end output. Additionally, 1% of PhiX, an adapter-ligated single-stranded DNA (ssDNA) virus, was spiked in the pooled libraries as a control in every sequencing run.

### Whole-genome *de novo* assembly.

The quality of the reads was assessed by Fastqc (http://www.bioinformatics.babraham.ac.uk/projects/fastqc/), and the adapter sequences and the sequences of poor quality were trimmed by Trim Galore (http://www.bioinformatics.babraham.ac.uk/projects/trim_galore/). Reads were assembled using assembler SPAdes v.3.1.1 (66) with default parameters, and the resulting contigs were filtered to be >500 bp and have a coverage of >4. We assessed the assemblage type of each isolate by mapping reads from each isolate to the reference genomes of G. duodenalis (assemblages A, B, and E) that were retrieved from the GiardiaDB (http://giardiadb.org) (67). To remove any potential contaminant contigs (non-*Giardia* reads), contigs were mapped to the NCBI nt database using BLASTn megablast (-best_hit_overhang 0.25 -best_hit_score_edge 0.05). If 80% of hits within two times the minimum E value mapped to G. duodenalis genomes, the contigs were kept. Additionally, any contigs that did not fit this criterion were mapped to a combined reference of assemblage A (WB) and assemblage B (GS_B) using BLASTn megablast with the same parameters, and those for which >500 bp matched the reference were added to the final contig set.

Even after removing contaminating reads, five samples had a total contig length greater than 14 Mb. This is significantly larger than the expected genome size of 10 to 11 Mb, suggesting further contamination from within *Giardia* species. Reads of these five samples were each mapped to a combined reference of assemblages A and B (WB, DH_A, and GS_B) and using BLASTn megablast, with the same parameters. In mixed samples, reads which hit only the minority assemblages (3.5 to 28.7% of total reads) were removed. SPAdes assembly and subsequent filtering were then performed on these filtered reads as described above. This resulted in two of the five samples having a combined contig length of <12,000,000 bp; however, three samples were still above this length and were excluded from analysis, as we could not separately the contamination from the major assemblage. Final contigs from each sample, along with the references WB and GS_B, were used to construct two assemblage specific pan-genomes using Panseq (68) with default parameters. The assembled contigs of each sample were arranged and compared using MUMmer (69).

### Whole-genome reference-based assembly and variant detection.

Reads were trimmed using cutadapt v1.8 (70) to remove Illumina adapters and reads with quality of <20 or length of <200. Assemblage assignment was then performed by mapping trimmed reads to a combined reference consisting of assemblage A (WB) and assemblage B (GS_B) using bowtie2 v2.1.0 (71) (with parameters --phred33 --local --dovetail --maxins 850) and determining the assemblage based on what the majority (70 to 99%) of reads mapped to. Trimmed reads were then remapped to the single majority assemblage using bowtie2 (--local).

Four samples had >5% of reads mapped to the minor assemblage after the initial bowtie2 alignment, so these were included in the analysis for both assemblages. For analysis in their minor assemblage, reads were mapped to a combined reference of WB, DH_A, and GS_B, using BLASTn megablast, with the same parameters as described above, and reads which hit only the majority assemblages were removed. The remaining reads were then included in the discoSNP analysis as below.

Single nucleotide variant (SNV) calling was performed by entering trimmed reads into discoSNP v2.1.2 (54) with parameters -c 4 –b 1 –k 31 –P 3, and a separate run for each assemblage. SNVs generated using discoSNP are output in “bubbles,” where each bubble consists of two distinct sequences (or branches) of *k* + 2 nodes, with the start and the end nodes in common. Each bubble was aligned to the assemblage-specific pan-genome using BLASTn and filtered so that only bubbles that hit a single unique position in the pan-genome were considered for further analysis. Additionally, bubbles were removed if any sample had a combined coverage over both branches of the bubble of <4 or any sample had <75% coverage in the majority branch. Filtered discoSNP output was then converted into a FASTA file using the SNV base from the branch with the highest coverage from each bubble. Phylogenetic trees were generated using FastTree v2.1.8 (default parameters) (72) and drawn using FigTree v1.4.0 (http://tree.bio.ed.ac.uk/software/figtree/). The phylogenetic relationships among isolates were also inferred using SplitsTree, and a Φ-test was conducted to estimate the level of recombination (73). The correlation between genetic and geographic distances was established using MEGA (74) and GenAlEx (75).

### Accession number(s).

Genome assemblies and raw reads are available at NCBI under BioProject no. PRJNA280606.
