# Supplementary figures and images for: Beaver Fever: Whole-Genome Characterization of Waterborne Outbreak and Sporadic Isolates To Study the Zoonotic Transmission of Giardiasis
Source: mSphere. 2018 Apr 25;3(2):e00090-18. doi: 10.1128/mSphere.00090-18 (PMC5917422; doi:10.1128/mSphere.00090-18)

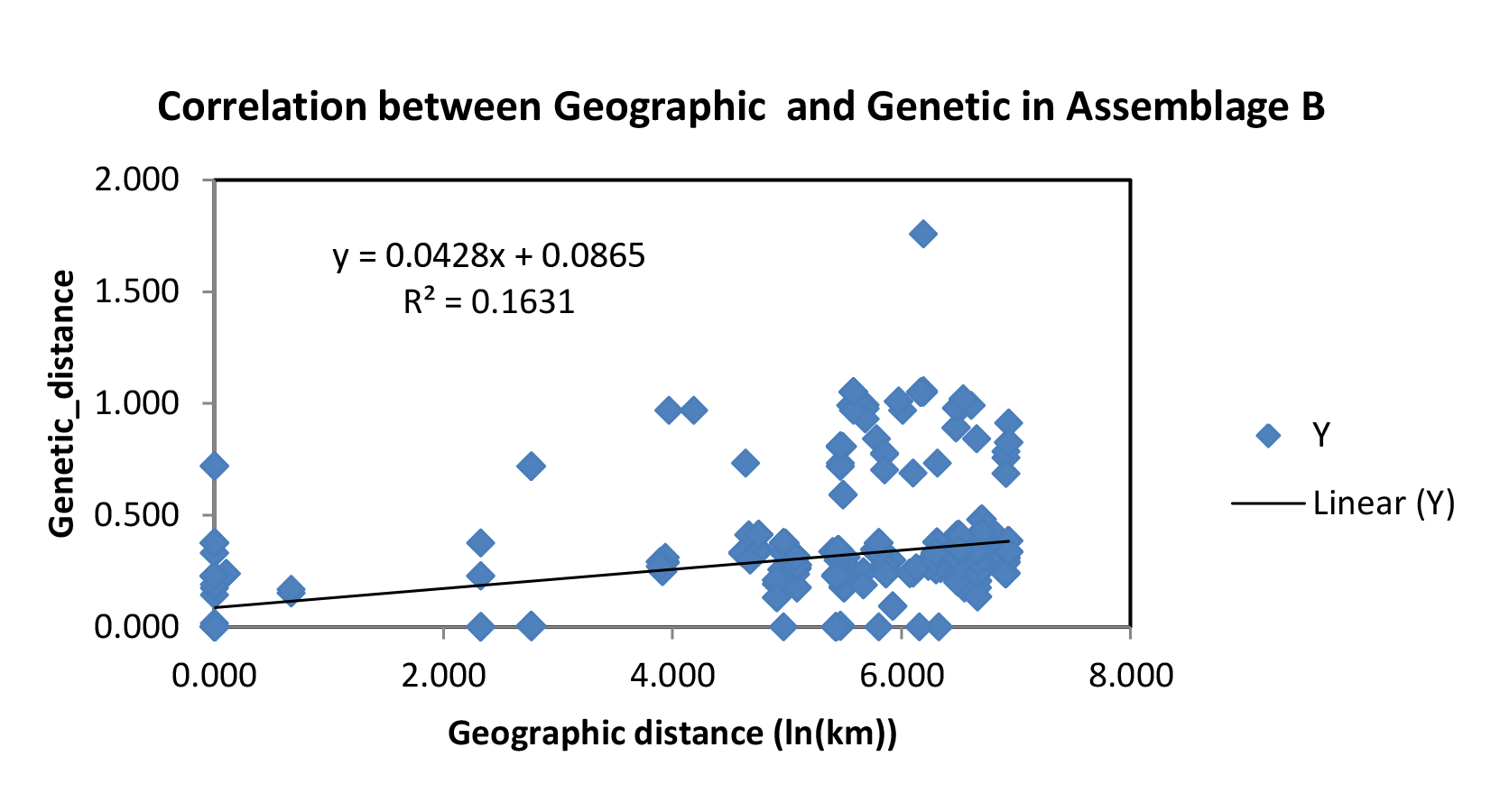

Supplement: FIG S1 [file sph002182525sf1.tif]
