# Supplementary material for: Beaver Fever: Whole-Genome Characterization of Waterborne Outbreak and Sporadic Isolates To Study the Zoonotic Transmission of Giardiasis
Source: mSphere. 2018 Apr 25;3(2):e00090-18. doi: 10.1128/mSphere.00090-18 (PMC5917422; doi:10.1128/mSphere.00090-18)
Supplement: FIG S2 [file sph002182525sf2.ppt]

## Slide 1
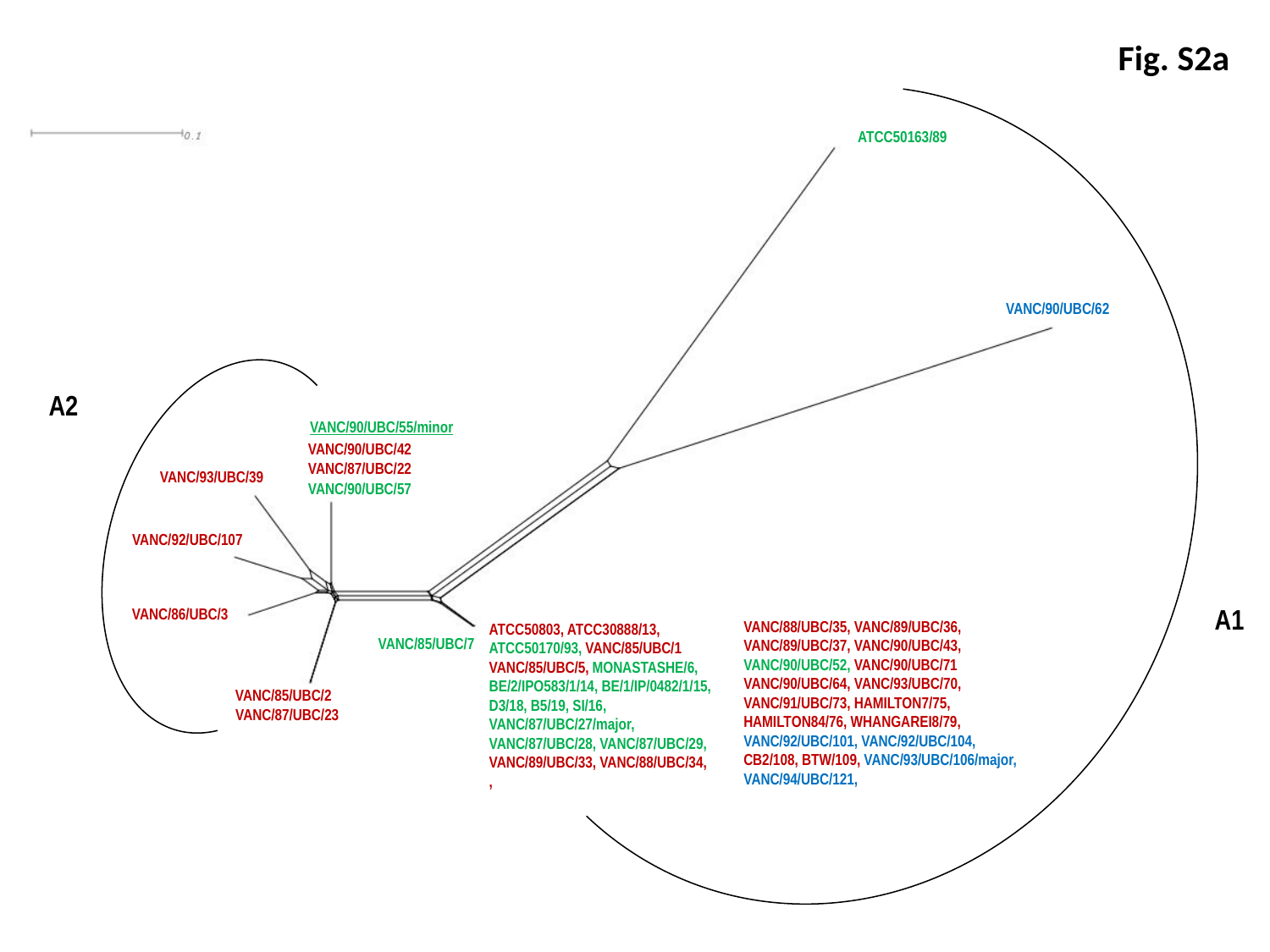

Fig. S2a
ATCC50163/89
VANC/90/UBC/62
A2
VANC/90/UBC/55/minor
VANC/90/UBC/42
VANC/87/UBC/22
VANC/90/UBC/57
VANC/93/UBC/39
VANC/92/UBC/107
A1
VANC/86/UBC/3
VANC/88/UBC/35, VANC/89/UBC/36,
VANC/89/UBC/37, VANC/90/UBC/43,
VANC/90/UBC/52, VANC/90/UBC/71
VANC/90/UBC/64, VANC/93/UBC/70,
VANC/91/UBC/73, HAMILTON7/75,
HAMILTON84/76, WHANGAREI8/79,
VANC/92/UBC/101, VANC/92/UBC/104,
CB2/108, BTW/109, VANC/93/UBC/106/major,
VANC/94/UBC/121,
ATCC50803, ATCC30888/13,
ATCC50170/93, VANC/85/UBC/1
VANC/85/UBC/5, MONASTASHE/6,
BE/2/IPO583/1/14, BE/1/IP/0482/1/15,
D3/18, B5/19, SI/16,
VANC/87/UBC/27/major,
VANC/87/UBC/28, VANC/87/UBC/29,
VANC/89/UBC/33, VANC/88/UBC/34,
,
VANC/85/UBC/7
VANC/85/UBC/2
VANC/87/UBC/23

## Slide 2
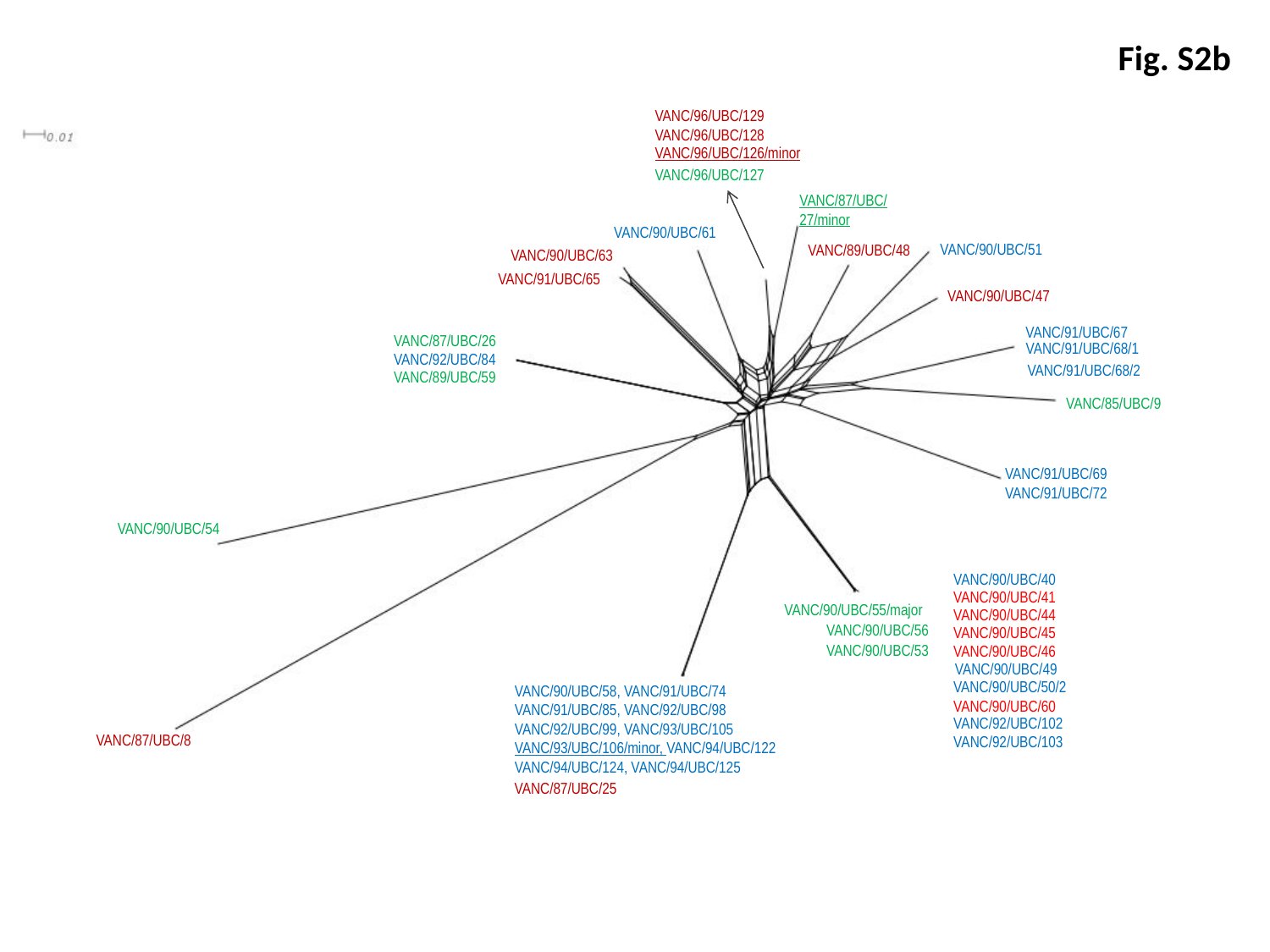

Fig. S2b
VANC/96/UBC/129
VANC/96/UBC/128
VANC/96/UBC/126/minor
VANC/96/UBC/127
VANC/87/UBC/
27/minor
VANC/90/UBC/61
VANC/90/UBC/51
VANC/89/UBC/48
VANC/90/UBC/63
VANC/91/UBC/65
VANC/90/UBC/47
VANC/91/UBC/67
VANC/87/UBC/26
VANC/91/UBC/68/1
VANC/92/UBC/84
VANC/91/UBC/68/2
VANC/89/UBC/59
VANC/85/UBC/9
VANC/91/UBC/69
VANC/91/UBC/72
VANC/90/UBC/54
VANC/90/UBC/40
VANC/90/UBC/41
VANC/90/UBC/55/major
VANC/90/UBC/44
VANC/90/UBC/56
VANC/90/UBC/45
VANC/90/UBC/53
VANC/90/UBC/46
VANC/90/UBC/49
VANC/90/UBC/50/2
VANC/90/UBC/58, VANC/91/UBC/74
VANC/91/UBC/85, VANC/92/UBC/98
VANC/92/UBC/99, VANC/93/UBC/105
VANC/93/UBC/106/minor, VANC/94/UBC/122
VANC/94/UBC/124, VANC/94/UBC/125
VANC/90/UBC/60
VANC/92/UBC/102
VANC/87/UBC/8
VANC/92/UBC/103
VANC/87/UBC/25
